# Disparities in Travel-Related Barriers to Accessing Health Care From the 2017 National Household Travel Survey

**DOI:** 10.1001/jamanetworkopen.2023.25291

**Published:** 2023-07-27

**Authors:** Muhieddine Labban, Chang-Rong Chen, Nicola Frego, David-Dan Nguyen, Stuart R. Lipsitz, Amanda J. Reich, Timothy R. Rebbeck, Toni K. Choueiri, Adam S. Kibel, Hari S. Iyer, Quoc-Dien Trinh

**Affiliations:** 1 Division of Urological Surgery, Brigham and Women’s Hospital, Harvard Medical School, Boston, Massachusetts; 2 Center for Surgery and Public Health, Brigham and Women’s Hospital, Harvard Medical School, Boston, Massachusetts; 3 Vita-Salute San Raffaele University, Milan, Italy; 4 Department of Urology, IRCCS Humanitas Research Hospital, Milan, Italy; 5 Division of Urology, University of Toronto, Toronto, Ontario, Canada; 6 Division of Population Sciences, Dana-Farber Cancer Institute, Boston, Massachusetts; 7 Department of Epidemiology and Zhu Family Center for Global Cancer Prevention, Harvard T.H. Chan School of Public Health, Boston, Massachusetts; 8 Lank Center for Genitourinary Oncology, Dana-Farber Cancer Institute, Boston, Massachusetts; 9 Section of Cancer Epidemiology and Health Outcomes, Rutgers Cancer Institute of New Jersey, New Brunswick

## Abstract

**Importance:**

Geographic access, including mode of transportation, to health care facilities remains understudied.

**Objective:**

To identify sociodemographic factors associated with public vs private transportation use to access health care and identify the respondent, trip, and community factors associated with longer distance and time traveled for health care visits.

**Design, Setting, and Participants:**

This cross-sectional study used data from the 2017 National Household Travel Survey, including 16 760 trips or a nationally weighted estimate of 5 550 527 364 trips to seek care in the United States. Households that completed the recruitment and retrieval survey for all members aged 5 years and older were included. Data were analyzed between June and August 2022.

**Exposures:**

Mode of transportation (private vs public transportation) used to seek care.

**Main Outcomes and Measures:**

Survey-weighted multivariable logistic regression models were used to identify factors associated with public vs private transportation and self-reported distance and travel time. Then, for each income category, an interaction term of race and ethnicity with type of transportation was used to estimate the specific increase in travel burden associated with using public transportation compared a private vehicle for each race category.

**Results:**

The sample included 12 092 households and 15 063 respondents (8500 respondents [56.4%] aged 51-75 years; 8930 [59.3%] females) who had trips for medical care, of whom 1028 respondents (6.9%) were Hispanic, 1164 respondents (7.8%) were non-Hispanic Black, and 11 957 respondents (79.7%) were non-Hispanic White. Factors associated with public transportation use included non-Hispanic Black race (compared with non-Hispanic White: adjusted odds ratio [aOR], 3.54 [95% CI, 1.90-6.61]; *P* < .001) and household income less than $25 000 (compared with ≥$100 000: aOR, 7.16 [95% CI, 3.50-14.68]; *P* < .001). The additional travel time associated with use of public transportation compared with private vehicle use varied by race and household income, with non-Hispanic Black respondents with income of $25 000 to $49 999 experiencing higher burden associated with public transportation (mean difference, 81.9 [95% CI, 48.5-115.3] minutes) than non-Hispanic White respondents with similar income (mean difference, 25.5 [95% CI, 17.5-33.5] minutes; *P* < .001).

**Conclusions and Relevance:**

These findings suggest that certain racial, ethnic, and socioeconomically disadvantaged populations rely on public transportation to seek health care and that reducing delays associated with public transportation could improve care for these patients.

## Key Points

**Question:**

What sociodemographic factors are associated with geographic barriers in accessing health care facilities in the United States?

**Findings:**

In this cross-sectional study including 12 092 households and 15 063 respondents, non-Hispanic Black respondents and economically disadvantaged populations were more likely to use public transportation. Among respondents with an income of $25 000 to $49 999, non-Hispanic Black respondents had longer trip duration than non-Hispanic White respondents, despite a similar distance traveled.

**Meaning:**

These findings suggest that achieving health equity in access to care will require the removal of access barriers that disproportionately affect socioeconomically disadvantaged and racially minoritized populations.

## Teaser

This cross-sectional study examines sociodemographic factors associated with public vs private transportation use to access health care and respondent, trip, and community factors associated with longer distance and time traveled for health care visits.

## Introduction

Achieving health equity requires the removal of access barriers that disproportionately impact socioeconomically disadvantaged and racially minoritized populations, such as Black and Hispanic individuals.^1,2,3,4^ Among these barriers, geographic access, referring to the proximity and time needed to travel from residence to facility, remains understudied relative to other dimensions of access.^5^

In the US, an estimated 5.8 million individuals delay medical care every year due to transportation barriers, including lack of a private vehicle; inconvenient, unreliable, and expensive transportation; and poor road infrastructure.^6,7^ Racially minoritized populations, patients with lower socioeconomic status, and patients with comorbidities are more likely to face transportation barriers and longer travel times for medical care.^6,8^ Discriminatory housing policies and resulting neighborhood segregation have placed racially minoritized populations far from high-quality care and reliable transport networks.^4,9,10^ Inadequate transportation can lead to missed or rescheduled appointments, delayed care, and poor medication adherence.^11,12,13,14^

We have a limited understanding of the potential impact of transportation modes on disparities in accessing care. Black populations are 3 times and Hispanic populations are 2 times more likely to use public transportation compared with White populations.^15^ These patterns align with disparities in car ownership, as 24% of Black and 17% of Hispanic households do not have a private vehicle, compared with only 7% of White households.^16^ Consequently, racially minoritized households with lower incomes may face compounded disadvantages, potentially leading to worse medical outcomes.^17,18^ For instance, neighborhoods with lower vehicle ownership are associated with higher mortality rates after myocardial infarction among Black patients, although disparities are mitigated in neighborhoods with higher rates of vehicle ownership.^19^ Similarly, residence in redlined areas is associated with worse breast cancer outcomes.^20^ More recently, the COVID-19 pandemic further widened this gap, due to safety concerns associated with public transportation use and lower access to telehealth among certain racial and ethnic groups.^21,22^

To improve understanding of barriers to accessing health care, we sought to identify sociodemographic factors associated with public vs private transportation as well as longer distance and time traveled for health care visits. We hypothesized that compared with non-Hispanic White respondents, non-Hispanic Black respondents are more likely to use public transportation and experience longer travel time to seek medical care, with the largest disparities observed among those with lower household incomes.

## Methods

### Study Design and Data Source

We conducted a cross-sectional study using the US Department of Transportation’s 2017 National Household Travel Survey (NHTS).^23^ Since the survey uses deidentified data, we obtained a human participants exemption from review and informed consent from the Mass General Brigham institutional review board. The study was carried in accordance with relevant guidelines and regulations with the Declaration of Helsinki. Informed consent to participate in the survey was obtained by the US Department of Transportation. This nationally representative data set serves as the primary source of information on daily travel behavior and modes of transportation.^23^ The data were analyzed between June and August 2022. This study adheres to the Strengthening the Reporting of Observational Studies in Epidemiology (STROBE) reporting guideline for observational studies.

### Sampling Design and Sample Weighting

The NHTS was conducted from March to May 2017 among a stratified random address-based sampling of households across 50 states.^23^ The sampling frame was derived from the US Postal Service Computerized Delivery Sequence File, and addresses were assigned to 1 of the following 4 sampling strata: addresses located in Metropolitan Statistical Areas (MSAs) with access to heavy rail transit and more than 1 million people, MSAs with 1 million people but without access to heavy rail transit, MSAs with fewer than 1 million people, and non-MSAs. A sample of addresses were selected at 2 different periods throughout the recruitment process to ensure the sampling is nationally representative.

The first phase of the study involved the household recruitment survey, while the second phase of the study was a person-level survey. The primary mode of household recruitment involved a mail-back survey with phone or web responses as secondary options. The collection of person-level information was conducted via phone or web. The 2017 NHTS includes households that completed the household recruitment survey and retrieval survey for all members aged 5 years and older. Of 252 304 recruited households, the final data set included information from 129 696 households, 264 234 respondents, and 923 572 trips over a year. The weighted recruitment response rate, which accounts for base weights representing the probability of selection for each sampled address, was 30.4% The weighted survey retrieval rate was 51.4%. The overall response rate, the product of the recruitment and retrieval response rates, was 15.6%. Weighted response rates were used to account for regional sampling biases. To aid data collection, households completing the recruitment interview were provided with personalized travel logs to record their travel on the assigned day. Informants recorded a 24-hour travel diary for days assigned randomly throughout the week between April 19, 2016, and April 25, 2017. A respondent recruitment flowchart is included in eFigure in Supplement 1.

The survey data were weighted over 12 months, generating annual national estimates. Weights were adjusted for household-level nonresponse by accounting for characteristics that were known to be associated with response propensity.^24^ Iterative proportional fitting was used to ensure that the final weights sum to known benchmark controls from the American Community Survey adjusting for nonresponse and population undercoverage, including geography and race and ethnicity.^24^

### Population

The survey included households with noninstitutionalized civilians with at least 1 adult household member. Trips for household members who were aged 5 years and older were included. We restricted the sample to trips for health care visits (medical, dental, and therapy). The type of health care visit was not recorded in the NHTS.

### Measures

Our primary comparison was the use of public transportation vs private vehicle. Public transportation was defined as the use of 1 of the following modes of transportation: public or commuter bus, intercity or commuter rail, and subway, elevated, light rail, or streetcar. Private transportation was defined as the use of 1 of the following modes of transportation: car, SUV, van, and pickup truck. We excluded trips made with taxis and rideshares because they constituted only 0.73% of all health care trips.

Respondents used online mapping software (Google Maps; Alphabet) to map their trip origin and destination and for each trip specified the trip purpose, mode of transportation, and time of the day. To estimate the burden of travel experienced for health care visits, we used the respondent-reported trip duration and the calculated shortest path distance generated by the mapping software’s application programming interface, based on mapped trip origin and trip distance.

### Covariates

We categorized covariates into person-, trip-, and community-level correlates selected based on prior literature.^6,25^ We also examined self-reported race and ethnicity and annual household income quartiles as modifiers of associations between public transportation use and travel burden.

Self-reported race and ethnicity were categorized into Hispanic, non-Hispanic Black, non-Hispanic White, and other (including American Indian or Alaska Native, Asian, Native Hawaiian or other Pacific Islander, and respondents with multiple races.). Additional person-level covariates included sex, age (<26, 26-50, 51-75, and ≥76 years), highest educational attainment (≤high school graduate; some college, associate degree, or bachelor’s degree; and graduate degree or professional degree), and household annual income quartiles (<$25 000, $25 000-$49 999, $50 000-$999 999, and ≥$100 000). Trip-level covariates included the use of public transport, trip on business days (Monday through Friday), and trip during business hours (between 8:00 am and 4:59 pm). Community-level covariates included household urban area classification based on the 2014 TIGER/Line Shapefile (urban, urbanized area or urban cluster, and rural), US Census region (Northeast, Midwest, South, and West), household located in an area with heavy metropolitan rail or subway, and proportion of renter-occupied housing in the census tract of the household’s location.

### Statistical Analysis

We used the trip as the unit of analysis. We weighed our sample using household identification number (ID) as the primary sampling unit to estimate outcomes and mean travel burden at the national level. We computed the median and IQR for the survey-weighted trip duration (minutes) and distance (miles) and compared them across covariates using Somers *D* test, an alternative to the Wilcoxon test for analyzing complex survey data.

To identify whether there are racial differences in public transportation use for health care visits, we fit a multivariable survey-weighted logistic regression adjusting for the respondent, trip, and community characteristics and clustered by household ID. Then, to assess factors associated with travel burden, we fit multivariable survey-weighted linear regression models separately for trip duration and distance. Models were adjusted for the respondent, trip, and community characteristics and clustered by household ID.

We performed subgroup analyses for trips that originated from urban vs rural settings. We presented results from models to estimate public transportation use in the overall population and urban only, due to few rural public transport users. Since socioeconomic status is associated with public transportation use,^26^ we divided our cohort by household annual income quartile. We assessed whether there was an interaction of race and ethnicity with public transportation use for trip time and distance in each income quartile. We conducted survey-weighted multivariable linear regression models, clustered by household ID, with a multiplicative interaction term for race and ethnicity and public transportation use. These models estimated the incremental change in mean trip time and distance for each race and ethnicity category when using public transportation compared with private vehicles. We repeated this analysis within each household income quartile.

All analyses were performed using Stata statistical software version 17 (StataCorp). *P* values were 2-sided, and *P* < .05 was considered significant.

## Results

### Trip Distribution by Respondent, Trip, and Community Characteristics

The sample included 12 092 households and 15 063 respondents who had trips for medical care, of whom 1028 respondents (6.9%) were Hispanic, 1164 respondents (7.8%) were non-Hispanic Black, and 11 957 respondents (79.7%) . Most respondents were female (8930 respondents [59.3%]), and 8500 respondents (56.4%) were aged 51 to 75 years. We captured 16 760 health care visit trips or a weighted annual national estimate of 5 550 527 364 trips, of which 15.0% (95%CI, 12.9%-17.3%) were taken by Hispanic individuals, 13.9% (95% CI, 11.8%-16.3%) were taken by non-Hispanic Black individuals, and 64.0% (95CI, 61.1%-66.8%) were taken by non-Hispanic White individuals. Most health care trips were made using a private vehicle (94.0% [95% CI, 93.3%-95.5%]) and originated in urban settings (81.1% [95% CI, 78.9%-83.2%]). Other respondent, trip, and community characteristics are depicted in Table 1.

**Table 1.** Travel Time and Distance by Respondent, Trip, and Community Characteristics, National Household Transportation Survey 2017

Characteristic	Trips, weighted % (95% CI) (N = 16 760)	Time, min		Distance, mi		Respondents total, No. (%) (N = 15 063)
		Median (IQR)	*P* value^a^	Median (IQR)	*P* value^a^	
Respondent level (n = 15 063)						
Sex						
Male	39.1 (37.2-41.0)	20.0 (10.0-30.0)	.24	5.8 (2.3-13.4)	.01	6133 (40.7)
Female	60.9 (59.0-62.9)	20.0 (10.0-30.0)		5.3 (2.0-11.1)		8930 (59.3)
Race						
Hispanic	15.0 (12.9-17.3)	20.0 (10.0-35.0)		5.3 (1.8-12.4)		1028 (6.9)
non-Hispanic Black	13.9 (11.8-16.3)	22.0 (13.0-40.0)	.07	4.3 (1.6-9.8)	.01	1164 (7.8)
Non-Hispanic White	64.0 (61.1-66.8)	20.0 (10.0-30.0)		5.8 (2.3-12.7)		11 957 (79.7)
Other^b^	7.1 (5.7-8.8)	19.0 (10.0-30.0)		5.0 (2.6-10.4)		854 (5.7)
Age, y						
≤25	14.6 (12.8-16.6)	18.0 (10.0-30.0)	<.001	5.6 (1.8-11.4)	.06	1122 (7.4)
26-50	30.3 (28.3-32.3)	16.0 (10.0-30.0)		5.2 (1.9-11.3)		3069 (20.4)
51-75	45.0 (42.6-47.4)	20.0 (15.0-34.0)		5.5 (2.3-11.7)		8500 (56.4)
≥76	10.1 (8.9-11.3)	20.0 (15.0-33.0)		5.8 (2.4-11.2)		2372 (15.8)
Education						
≤High school	34.8 (32.6-37.2)	20.0 (10.0-33.0)	.01	4.8 (2.0-11.3)	.33	3942 (27.1)
Some college or bachelor’s degree	48.8 (46.6-51.1)	20.0 (11.0-30.0		6.0 (2.3-12.0)		7564 (52.1)
Graduate or professional	16.4 (15.0-17.8)	17.0 (10.0-30.0)		4.9 (2.1-11.9)		3026 (20.8)
Income, $						
<25 000	27.3 (24.8-29.9)	25.0 (15.0-39.0)	<.001	4.7 (1.9-11.1)	.27	2961 (20.3)
25 000-49 999	23.3 (20.7-26.2)	20.0 (10.0-30.0)		6.2 (2.1-12.5)		3227 (22.2)
50 000-999 999	24.1 (22.1-26.2)	18.0 (10.0-30.0)		5.5 (2.2-12.8)		4490 (30.8)
≥100 000	25.3 (23.1-27.7)	15.0 (10.0-30.0)		5.5 (2.4-10.9)		3896 (26.7)
Trip level						
Transportation						
Private vehicle	94.0 (93.3-95.5)	20.0 (10.0-30.0)	<.001	6.0 (2.5-12.9)	<.001	14 730 (97.8)
Public	6.0 (4.5-6.7)	50.0 (28.0-75.0)		3.9 (2.1-7.6)		333 (2.2)
Day of the week						
Business days	92.3 (90.8-93.5)	20.0 (10.0-30.0)	.89	5.5 (2.2-11.7)	.62	14 364 (95.4)
Weekends	7.7 (6.5-9.2)	20.0 (10.0-30.0)		5.0 (2.1-11.1)		699 (4.6)
Time of the day						
Off hours	15.4 (13.7-17.2)	21.0 (15.0-40.0)	.002	6.2 (2.7-13.8)	.002	2005 (13.3)
Business hours	84.6 (82.8-86.3)	20.0 (10.0-30.0)		5.3 (2.0-11.4)		13 058 (86.7)
Community level						
Urban or rural						
Urban	81.1 (78.9-83.2)	18.0 (10.0-30.0)	<.001	4.6 (2.0-9.8)	<.001	11 449 (76.0)
Rural	18.9 (16.8-21.1)	27.0 (15.0-40.0)		12.2 (4.6-20.8)		3614 (24.0)
Region						
Northeast	18.7 (16.6-20.9)	20.0 (11.0-30.0)	.78	4.1 (1.7-9.2)	<.001	2355 (15.6)
Midwest	21.2 (18.9-23.8)	20.0 (10.0-30.0)		5.5 (2.3-11.3)		2207 (14.7)
South	38.1 (35.4-40.8)	20.0 (10.0-34.0)		6.1 (2.1-14.7)		6870 (45.6)
West	22.0 (20.1-24.1)	20.0 (10.0-30.0)		5.7 (2.4-11.1)		3631 (24.1)
Railway nearby						
Yes	27.9 (25.7-30.4)	20.0 (11.0-30.0)	.11	4.3 (1.9-9.0)	<.001	2197 (14.6)
No	72.1 (69.6-74.3)	20.0 (10.0-30.0)		5.9 (2.2-13.4)		12 866 (85.4)
Renters, %						
<25	41.7 (39.2-44.2)	20.0 (14.0-31.0)	.78	7.8 (3.4-15.6)	<.001	6776 (45.0)
25-54	43.2 (40.7-45.8)	15.0 (10.0-30.0)		4.3 (1.7-9.6)		6292 (41.8)
≥55	15.1 (13.6-16.7)	20.0 (10.0-30.0)		3.4 (1.4-8.3)		1979 (13.2)

^a^ Since trip time and distance are not normally distributed, Somers *D* was used to estimate the survey-weighted difference in trip time and distance across the covariates as both trip time and distance.

^b^ Includes American Indian or Alaska Native, Asian, Native Hawaiian or other Pacific Islander, and respondents with multiple races.

#### Unadjusted Median Trip Duration and Distance by Respondent, Trip, and Community Characteristics

Non-Hispanic Black respondents traveled shorter distances compared with non-Hispanic White respondents (median [IQR], 4.3 [1.6-9.8] miles vs 5.8 [2.3-12.7] miles; *P* = .01) but had similar median travel times (median [IQR] duration, 22.0 [13.0 to 40.0] minutes vs 20.0 [10.0 to 30.0] minutes; *P* = .07). Respondents with a household income less than $25 000 had longer trip durations than respondents with a household income of $100 000 or more (median [IQR] duration, 25.0 [15.0 to 39.0] minutes vs 15.0 [10.0 to 30.0] minutes; *P* < .001) but traveled similar distances. Although trips using public transport were shorter in distance (median [IQR] distance, 3.9 [2.1 to 7.6] miles vs 5.9 [2.5 to 12.9] miles; *P* < .001), they were more than twice as long in duration compared with trips in private vehicles (median [IQR] duration, 50.0 [28.0 to 75.0] minutes vs 20.0 [10.0 to 30.0] minutes; *P* < .001). The median trip times and distances by respondent, trip, and community characteristics are illustrated in Table 1.

#### Factors Associated With Public Transportation Use

Among the entire cohort, non-Hispanic Black respondents had increased odds of using public transportation for health care visits compared with non-Hispanic White respondents (odds ratio [OR], 3.54 [95% CI, 1.90 to 6.61]; *P* < .001) (Table 2). Additionally, compared with respondents with household income of $100 000 or greater, respondents with household income less than $25 000 were more likely to use public transportation (OR, 7.16 [95% CI, 3.50 to 14.68]; *P* < .001).

**Table 2.** Survey-Weighted Multivariable Logistic Regressions for Factors Associated With Public vs Private Transport Use for Health Care Visits, 2017 National Household Transportation Survey

Factor	Public transportation use			
	Overall cohort^a^ (N = 14 810; WE = 4 635 469 286)		Respondents residing in urban setting^a^ (N = 11 135; WE = 3 710 436 381)	
	aOR (95%CI)	*P* value	aOR (95%CI)	*P* value
Race				
Hispanic	1.70 (0.85-3.38)	.13	1.68 (0.84-3.35)	.14
Non-Hispanic Black	3.54 (1.90-6.61)	<.001	3.50 (1.87-6.54)	<.001
Non-Hispanic White	1 [Reference]	NA	1 [Reference]	NA
Other^b^	1.60 (0.69-3.72)	.28	1.58 (0.68-3.71)	.29
Age, y				
<26	1 [Reference]	NA	1 [Reference]	NA
26-50	1.05 (0.47-2.32)	.91	1.03 (0.46-2.30)	.93
51-75	0.92 (0.37-2.26)	.85	0.89 (0.36-2.23)	.81
≥76	0.49 (0.15-1.62)	.24	0.49 (0.15-1.60)	.24
Sex				
Male	1 [Reference]	NA	1 [Reference]	NA
Female	0.93 (0.60-1.44)	.74	0.92 (0.59-1.43)	.71
Education				
Graduate	1 [Reference]	NA	1 [Reference]	NA
Some college or bachelor’s degree	0.99 (0.48-2.04)	.98	0.99 (0.45-2.03)	.97
≤High school	2.41 (1.13-5.15)	.02	2.41 (1.12-5.15)	.02
Household income, $				
≥100 000	1 [Reference]	NA	1 [Reference]	NA
50 000-99 999	2.18 (1.02-4.63)	.04	2.21 (1.03-4.72)	.04
25 000-49 999	1.30 (0.53-3.19)	.57	1.30 (0.52-3.22)	.58
<25 000	7.16 (3.50-14.68)	<.001	7.28 (3.53-15.0)	<.001
Medical condition limiting driving	2.43 (1.24-4.79)	.01	2.46 (1.24-4.88)	.01
Family size				
≤2	1 [Reference]	NA	1 [Reference]	NA
≥3	0.71 (0.37-1.34)	.29	0.72 (0.38-1.36)	.31
Trip characteristics				
Day of the week				
Business days	1 [Reference]	NA	1 [Reference]	NA
Weekend	0.82 (0.39-1.72)	.60	0.83 (0.39-1.74)	.62
Time of the day				
Off hours	1 [Reference]	NA	1 [Reference]	NA
Business hours	0.45 (0.27-0.76)	.003	0.46 (0.27-0.77)	.004
Community-level characteristics				
Near railway	0.21 (0.12-0.37)	<.001	0.21 (0.12-0.38)	<.001
Region				
Northeast	1 [Reference]	NA	1 [Reference]	NA
Midwest	0.31 (0.13-0.74)	.009	0.31 (0.13-0.75)	.01
South	0.25 (0.13-0.49)	<.001	0.25 (0.13-0.49)	<.001
West	0.31 (0.17-0.58)	<.001	0.31 (0.17-0.58)	<.001
Renters, %				
<25	1 [Reference]	NA	1 [Reference]	NA
25-54	7.43 (3.77-14.64)	<.001	7.51 (3.76-15.0)	<.001
55-100	10.76 (5.20-22.25)	<.001	10.80 (5.17-22.67)	<.001
Residence				
Urban	1 [Reference]	NA	NA	NA
Rural	0.07 (0.03-0.19)	<.001	NA	NA

Abbreviations: aOR, adjusted odds ratio; NA, not applicable; WE, weighted national estimate.

^a^ Models were adjusted for sex, race and ethnicity, age group, educational attainment, household income, medical condition limiting driving, family size, whether the trip was made on business days and business hours, US region, whether the place of residence was close to railway, the proportion of renters in the neighborhood, and whether the residence was in an urban or rural setting.

^b^ Includes American Indian or Alaska Native, Asian, Native Hawaiian or other Pacific Islander, and respondents with multiple races.

Respondents with no access to railway (OR, 0.21 [95% CI, 0.12 to 0.74]; *P* < .001) and respondents residing in rural settings (OR, 0.07 [95% CI, 0.03 to 0.19]; *P* < .001) reported lower odds of public transportation use; while respondents residing in areas with more than 55% of renter-occupied households had higher odds of public transportation use (OR, 10.76 [95% CI, 5.20 to 22.25]; *P* < .001). Additionally, respondents residing in the South, Midwest, and West census regions were less likely than those in the Northeast to use public transportation for health visit trips (Table 2). We also found similar results among respondents residing in urban settings (Table 2).

#### Correlates Associated With Longer Health Visit Trip Duration and Distance

Table 3 depicts the factors associated with longer trip duration and Table 4 depicts the factors associated with longer trip distance. Longer trip duration was observed among non-Hispanic Black respondents compared with White respondents in urban settings (mean difference, 4.8 [95% CI, 0.01 to 9.5] minutes), but not in rural settings (Table 3). No differences in trip distance were observed by race and ethnicity (Table 3). Despite traveling a similar median distance, respondents with a household income less than $25 000 spent a mean difference of 5.1 (95% CI, 1.8 to 8.3) minutes longer than respondents with a household income of $100 000 or more. Respondents with household income less than $25 000, compared with respondents with household income of $100 000 or more, in rural settings traveled longer distances (mean difference, 6.2 [95% CI, 0.8 to 11.6] miles) and for longer durations (mean difference, 13.8 [95% CI, 5.9 to 22.3] minutes).

**Table 3.** Survey-Weighted Multivariable Linear Regression for Time Traveled for Health Care Visits at the National Level and in Urban and Rural Settings, 2017 National Household Transportation Survey

Variables	Time, B (95%CI), min		
	Overall trips (N = 14 811; WE = 4 635 520 357)	Trips from urban setting (n = 11 135; WE = 3 710 436 381)^a^	Trips from rural setting (n = 3676; WE = 925 083 977)^a^
Respondent characteristics			
Sex			
Male	0 [Reference]	0 [Reference]	0 [Reference]
Female	−2.4 (−4.5 to −0.4)	−2.4 (−4.5 to −0.3)	−2.7 (−8.3 to 2.8)
Race			
Hispanic	1.8 (−1.9 to 5.4)	1.3 (−2.3 to 4.9)	6.8 (−8.9 to 22.5)
Non-Hispanic Black	4.7 (−0.1 to 9.4)	4.8 (<0.1 to 9.5)	1.9 (−19.6 to 23.4)
Non-Hispanic White	0 [Reference]	0 [Reference]	0 [Reference]
Other^b^	−2.7 (−6.1 to 0.7)	−1.8 (−5.2 to 1.6)	−12.1 (−25.1 to 0.9)
Age, y			
<26	0 [Reference]	0 [Reference]	0 [Reference]
26-50	3.1 (0.2 to 5.9)	1.1 (−1.8 to 3.9)	11.1 (1.9 to 20.2)
51-75	6.8 (3.9 to 9.6)	4.6 (1.8 to 7.4)	15.1 (6.0 to 24.3)
≥76	6.4 (2.8 to 10.1)	4.3 (0.8 to 7.8)	14.8 (3.6 to 26.1)
Education			
Graduate	0 [Reference]	0 [Reference]	0 [Reference]
Some college or bachelor’s degree	−0.6 (−3.7 to 2.5)	0.2 (−2.2 to 2.6)	−6.0 (−15.2 to 3.1)
≤High school	−0.4 (−2.9 to 2.1)	−0.7 (−3.6 to 2.1)	−2.8 (−12.9 to 7.4)
Income, $			
≥100 000	0 [Reference]	0 [Reference]	0 [Reference]
50 000-99 999	2.4 (−0.2 to 5.0)	0.7 (−1.7 to 3.1)	10.5 (2.3 to 18.8)
25 000-49 999	2.8 (−0.1 to 5.8)	1.9 (−1.2 to 5.0)	9.4 (2.5 to 16.3)
<25 000	5.1 (1.8 to 8.3)	3.8 (0.3 to 7.2)	13.8 (5.3 to 22.3)
Trip characteristics			
Public transport	34.2 (26.5 to 41.8)	34.5 (27.0 to 42.1)	23.7 (−3.4 to 50.7)
Business days	−2.8 (−8.4 to 2.8)	−3.4 (−9.5 to 2.7)	2.6 (−11.3 to 16.5)
Business hours	−8.5 (−12.4 to −4.6)	−8.2 (−12.4 to −4.0)	−9.7 (−19.9 to 0.5)
Community characteristics			
Rural	11.1 (7.4 to 14.7)	NA	NA
Region			
Northeast	0 [Reference]	0 [Reference]	0 [Reference]
Midwest	−1.5 (−5.1 to 2.0)	−2.0 (−5.8 to 1.8)	1.6 (−7.0 to 10.1)
South	2.2 (−0.9 to 5.3)	2.6 (−0.8 to 6.0)	1.5 (−5.8 to 8.8)
West	3.3 (<−0.1 to 6.7)	2.4 (−1.0 to 5.7)	11.2 (−3.5 to 25.8)
Far from railway	−0.7 (−3.3 to 1.9)	−0.6 (−3.3 to 2.1)	0.4 (−11.3 to 12.1)
Renters, %			
<25	0 [Reference]	0 [Reference]	0 [Reference]
25-54	−3.4 (−5.8 to −1.0)	−2.8 (−5.1 to −0.5)	−4.6 (−12.9 to 3.6)
55-100	−3.9 (−7.0 to −0.8)	−3.3 (−6.3 to −0.4)	−6.0 (−25.0 to 12.9)

Abbreviations: NA, not applicable; WE, weighted national estimate.

^a^ Models were adjusted for sex, race and ethnicity, age group, educational attainment, household income, medical condition limiting driving, family size, whether the trip was made on business days and business hours, US region, whether the place of residence was close to railway, the proportion of renters in the neighborhood, and whether the residence was in an urban or rural setting.

^b^ Includes American Indian or Alaska Native, Asian, Native Hawaiian or other Pacific Islander, and respondents with multiple races.

**Table 4.** Survey-Weighted Multivariable Linear Regression for Distance Traveled for Health Care Visits at the National Level and in Urban and Rural Settings

Factor	Distance, B (95%CI), mi		
	Overall trips (N = 14 811; WE = 4 635 520 357)	Trips from urban setting (n = 11 135; WE = 3 710 436 381)^a^	Trips from rural setting (n = 3676; WE = 925 083 977)^a^
Respondent characteristics			
Sex			
Male	0 [Reference]	0 [Reference]	0 [Reference]
Female	−1.6 (−2.8 to −0.5)	−1.0 (−2.2 to 0.1)	−3.9 (−7.3 to −0.4)
Race			
Hispanic	2.1 (−0.5 to 4.7)	1.5 (−0.6 to 3.6)	7.0 (−9.0 to 23.1)
Non-Hispanic Black	2.0 (−0.5 to 4.4)	1.2 (−1.2 to 3.7)	5.1 (−5.9 to 16.1)
Non-Hispanic White	0 [Reference]	0 [Reference]	0 [Reference]
Other^b^	−0.2 (−2.5 to 2.2)	0.3 (−2.0 to 2.6)	−6.9 (−13.4 to −0.5)
Age, y			
<26	Ref	Ref	Ref
26-50	1.0 (−0.7 to 2.7)	−0.4 (−2.0 to 1.2)	7.5 (1.1 to 13.9)
51-75	2.3 (0.6 to 4.0)	0.4 (−1.1 to 2.0)	10.0 (3.7 to 16.2)
≥76	1.2 (−0.6 to 3.0)	−0.3 (−2.0 to 1.4)	7.8 (2.1 to 13.5)
Education			
Graduate	0 [Reference]	0 [Reference]	0 [Reference]
College/Bachelors	−0.8 (−2.8 to 1.3)	0.0 (−1.7 to 1.7)	−4.2 (−12.5 to 4.1)
Highschool or less	−1.7 (−4.0 to 0.6)	−2.0 (−3.9 to −0.02)	−2.2 (−11.0 to 6.6)
Income, $			
≥100 000	0 [Reference]	0 [Reference]	0 [Reference]
50 000-99 999	1.8 (−0.1 to 3.6)	0.6 (−1.0 to 2.2)	6.6 (0.4 to 12.8)
25 000-49 999	0.8 (−0.9 to 2.4)	<0.1 (−1.6 to 1.6)	5.5 (0.9 to 10.1)
<25 000	1.8 (−0.2 to 3.7)	1.0 (−1.0 to 3.0)	6.2 (0.8 to 11.6)
Trip characteristics			
Public transport	−0.6 (−3.3 to 2.2)	−0.4 (−3.2 to 2.4)	−7.0 (−21.0 to 7.1)
Business days	0.1 (−2.0 to 2.3)	−0.4 (−2.7 to 1.8)	4.3 (−2.5 to 11.2)
Business hours	−4.4 (−6.9 to −1.9)	−3.7 (−6.3 to −1.2)	−7.1 (−14.6 to 0.4)
Community characteristics			
Rural	8.2 (5.9 to 10.5)	NA	NA
Region			
Northeast	0 [Reference]	0 [Reference]	0 [Reference]
Midwest	0.0 (−1.1 to 2.5)	−0.2 (−1.7 to 1.3)	5.0 (−1.6 to 11.7)
South	1.7 (0.3 to 3.2)	1.9 (0.5 to 3.3)	1.9 (−2.3 to 6.1)
West	2.6 (0.9 to 4.2)	1.6 (0.3 to 2.9)	9.2 (−0.5 to 19.0)
Far from railway	1.3 (0.1 to 2.6)	1.4 (0.2 to 2.6)	0.8 (−5.8 to 7.5)
Renters, %			
<25	0 [Reference]	0 [Reference]	0 [Reference]
25-54	−2.6 (−4.1 to −1.2)	−1.5 (−2.8 to −0.1)	−6.1 (−10.6 to −1.6)
55-100	−4.6 (−6.1 to −3.1)	−3.6 (−5.1 to −2.1)	−8.8 (−14.3 to −3.3)

Abbreviations: NA, not applicable; WE, weighted national estimate.

^a^ Models were adjusted for sex, race and ethnicity, age group, educational attainment, household income, medical condition limiting driving, family size, whether the trip was made on business days and business hours, US region, whether the place of residence was close to railway, the proportion of renters in the neighborhood, and whether the residence was in an urban or rural setting.

^b^ Includes American Indian or Alaska Native, Asian, Native Hawaiian or other Pacific Islander, and respondents with multiple races.

Respondents in rural settings had longer trip duration (mean difference, 11.1 [95% CI, 7.4 to 14.7] minutes) and distance (mean difference, 8.2 [95% CI, 5.9 to 10.5] minutes). Respondents residing in the West and South census regions had longer trip distance than those residing in the Northeast but reported no difference in trip duration relative to other census regions (Table 4). Respondents residing in communities with higher renter-occupied households reported shorter trip durations and distances (Table 3 and Table 4).

#### Interactions of Race and Ethnicity, Household Income, and Public Transportation

The incremental change in travel burden associated with public transportation relative to private vehicle use varied more by race for trip duration than travel distance (Table 5). Non-Hispanic Black respondents had longer trip durations using public transport vs private vehicle in the $25 000 to $49 999 household income category (mean difference, 81.9 [95% CI, 48.5 to 115.3] minutes), compared with non-Hispanic White respondents (mean difference, 25.5 [95% CI, 17.5 to 33.5] minutes; *P* for interaction < .001). Hispanic respondents using public transport had longer trip duration (mean difference, 23.9 [95% CI, 13.2 to 34.6] minutes) but shorter distances (mean difference, −5.8 [95% CI, −9.7 to −2.0] miles) compared with private vehicle use in the $25 000 to $49 999 household income category (*P* for interaction < .001). A subgroup analysis among trips taken from urban settings showed similar results (eTable in Supplement 1).

**Table 5.** Survey-Weighted Multivariable Linear Regression for Additional Travel Time and Distance Associated With Use of Public Transportation vs Private Vehicle to for Health Care Visits by Household Income

Factor	Household income, $^a^							
	<25 000 (n = 2830 trips; WE = 1 192 038 241)		25 000-49 999 (n = 3360 trips; WE = 1 121 140 466)		50 000-99 999 (n = 4734 trips; WE = 1 152 528 980)		≥100 000 (3887 trips; WE = 1 169 812 670)	
	B (95% CI)	*P* for interaction	B (95% CI)	*P* for interaction	B (95% CI)	*P* for interaction	B (95% CI)	*P* for interaction
**Travel time, min**								
Hispanic	32.4 (14.2 to 50.6)	.91	23.9 (13.2 to 34.6)	<.001	67.5 (31.0 to 104.1)	<.001	−6.1 (−13.3 to 1.1)	<.001
Non-Hispanic Black	37.0 (−1.2 to 75.1)		81.9 (48.5 to 115.3)		−22.4 (−49.8 to 4.9)		−0.1 (−63.9 to 63.8)	
Non-Hispanic White	28.5 (10.3 to 46.6)		25.5 (17.5 to 33.5)		26.5 (17.2 to 35.9)		33.5 (12.3 to 54.7)	
Other^b^	29.2 (0.4 to 58.1)		9.9 (−1.0 to 20.8)		67.4 (58.8 to 76.1)		56.5 (33.2 to 79.7)	
**Travel distance, mi**								
Hispanic	−0.5 (−4.7 to 3.7)	.83	−5.8 (−9.7 to −2.0)	.03	1.0 (−6.7 to 8.6)	.12	−5.8 (−10.0 to −1.6)	.03
Non-Hispanic Black	1.1 (−9.5 to 11.7)		5.0 (−1.9 to 12.0)		−6.9 (−16.0 to 2.2)		4.1 (−16.6 to 24.9)	
Non-Hispanic White	−2.3 (−5.6 to 1.1)		−1.2 (−4.9 to 2.4)		−1.6 (−4.8 to 1.6)		5.2 (−4.0 to 14.4)	
Other^b^	−2.5 (−9.1 to 4.1)		−3.7 (−8.2 to 0.7)		5.9 (0.2 to 11.6)		3.1 (−4.0 to 10.1)	

Abbreviation: WE, weighted national estimate.

^a^ Models were adjusted for sex, race and ethnicity, age group, educational attainment, household income, medical condition limiting driving, family size, whether the trip was made on business days and business hours, US region, whether the place of residence was close to railway, the proportion of renters in the neighborhood, and whether the residence was in an urban or rural setting.

^b^ Includes American Indian or Alaska Native, Asian, Native Hawaiian or other Pacific Islander, and respondents with multiple races.

## Discussion

This cross-sectional study used a nationally representative survey of households in the US to examine the respondent-, trip-, and community-level factors associated with public transportation use and travel burden for health care visits. We found that non-Hispanic Black respondents and respondents with lower socioeconomic status were more likely to use public transportation than non-Hispanic White respondents with higher income. We observed variations in the additional travel time between public and private transportation based on race and ethnicity and household income. Specifically, in the second income quartile, the additional trip duration associated with public transportation was significantly higher among non-Hispanic Black respondents compared with non-Hispanic White respondents. However, within the lowest and highest income groups, racial disparities in travel burden were mitigated, resulting in comparable travel time for health care visits. These findings are especially relevant in urban settings. Our findings indicate that certain racial and ethnic groups among lower income populations are more likely to use public transportation, which incurs a higher travel burden when seeking medical care.

Based on the findings of a study by Probst et al^25^ that uses the 2001 NHTS survey, we can compare trends in racial and ethnic patterns of travel burden when seeking health care in 2001 vs 2017. There was a decline in public transportation use from 2.7% in 2001 to 2.2% in 2017.^25^ A study by Wolfe et al^6^ using data from the National Health Interview Survey found that the reporting of transportation barriers had increased from 1997 to 2017.^6^ Taken together, these studies suggest that although its use has declined over the past 16 years, public transportation remains an important means for socially disadvantaged populations to access health care.

We further examined how public transportation was associated with travel burden across racial and ethnic and income strata. We found that travel burden using public transportation vs private vehicle use was greater among Hispanic and non-Hispanic Black respondents than non-Hispanic White respondents among those with and income of $25 000 to $49 999 and $50 000 to $99 999, but not among those with the lowest and highest income categories. This suggests that the influence of public transportation on travel burden varied based on both race and ethnicity and income, with the difference being more pronounced among certain income groups. Minoritized populations are more likely to live in neighborhoods that are poorly served by public transportation and far from high-quality health care services,^11^ consequences of racial segregation arising from discriminatory housing poalicies.^27^ Additionally, car ownership is associated with significant financial burden, especially with increasing gas prices and substantial parking fees at treatment centers.^28^ For example, patients with cancer require more frequent health care visits, so physical barriers to care can have a direct association with their receipt, continuity, and quality of treatment and care.^29,30^ These data suggest that interventions to augment or strengthen existing public transportation along routes from underserved areas to health care facilities could serve as an equity-oriented approach to increasing health care access. Nevertheless, since rural settings have less or no public transportation access and are inherently less densely populated, high costs of public transportation infrastructure in rural settings may require alternative strategies to address racial and ethnic disparities in affordable transportation to health care.^31^ Our findings, along with earlier studies demonstrating that socioeconomically disadvantaged populations made greater use of public transportation and experienced greater physical barriers to accessing health services, provide evidence for policy makers and transportation planners. Transportation barriers experienced by disadvantaged populations could be addressed by subsidizing costs and improving convenience of travel to and from medical facilities. Examples of potential interventions include vouchers, ridesharing services, or increasing access to telehealth among minoritized and economically disadvantaged populations.^32,33^ Ridesharing services have been proposed because they can be scheduled as needed, use direct routes, are readily available in most urban areas, and cost less.^34,35,36^ However, evidence suggests that ridesharing services might only reduce missed appointment rates among populations with specific transportation needs^37^ or among those requiring multiple visits for cancer treatment completion.^38^ Furthermore, successful implementation of these interventions would necessitate appropriate funding, infrastructure, and coordination.

### Limitations

This study has some limitations. This is a cross-sectional survey relying on a sample of participants who agreed to provide data; therefore, findings may not be generalizable to all communities. However, this is a large, nationally representative survey in the US, providing variability in race and ethnicity, socioeconomic status, and geographic coverage. Second, the survey collects participant-reported travel times and a web-based mapping service calculated shortest network path distance, which may not reflect the exact route taken by respondents, potentially underestimating disparities in travel burden.^23^ Third, the survey does not capture individuals who do not have access to health care because of other barriers, such as insurance coverage. Moreover, the type of health care visit is not reported; thus, we could not distinguish between the reason of visit or whether respondents had several visits per year. Although we have examined multilevel factors associated with longer commute duration, qualitative studies could further help in understanding the transportation barriers minoritized populations face.

## Conclusion

This cross-sectional study found that minoritized populations and respondents with lower socioeconomic status were more likely to rely on public transportation than other groups, contributing to a higher travel burden in accessing care. Strengthening or augmenting existing transportation routes targeting underserved populations could reduce racial disparities in access to care and downstream health outcomes.

## Notes

Supplement 1. **eTable.** Survey-Weighted Multivariable Linear Regression for Additional Travel Time (minutes) and Distance (miles) Associated With Use of Public Transportation vs Private Vehicle From Trip Originating in the Urban Setting for Health Care Visits Reported by Household Income (<$25 000; $25,000-$49,999; $50,000-$99,999; and ≥$100,000) Accounting for Respondent, Trip, and Community Characteristics **eFigure.** Respondent Recruitment Flowchart

Supplement 2.
Data Sharing Statement
