# Supplementary material for: Disparities in Travel-Related Barriers to Accessing Health Care From the 2017 National Household Travel Survey
Source: JAMA Netw Open. 2023 Jul 27;6(7):e2325291. doi: 10.1001/jamanetworkopen.2023.25291 (PMC10375305; doi:10.1001/jamanetworkopen.2023.25291)
Supplement: Supplement 1. — eTable. Survey-Weighted Multivariable Linear Regression for Additional Travel Time (minutes) and Distance (miles) Associated With Use of Public Transportation vs Private Vehicle From Trip Originating in the Urban Setting for Health Care Visits Reported by Household Income (<$25 000; $25,000-$49,999; $50,000-$99,999; and ≥$100,000) Accounting for Respondent, Trip, and Community Characteristics eFigure. Respondent Recruitment Flowchart [file jamanetwopen-e2325291-s001.pdf]

Supplemental Online Content

Labban M, Chen CR, Frego N, et al. Disparities in travel-related barriers to accessing health care from the 2017 National Household Travel Survey. *JAMA Netw Open*. 2023;6(7):e2325291. doi:10.1001/jamanetworkopen.2023.25291

eTable. Survey-Weighted Multivariable Linear Regression for Additional Travel Time (minutes) and Distance (miles) Associated With Use of Public Transportation vs Private Vehicle From Trip Originating in the Urban Setting for Health Care Visits Reported by Household Income (<$25,000; $25,000-$49,999; $50,000-$99,999; and ≥ $100,000) Accounting for Respondent, Trip, and Community Characteristics

eFigure. Respondent Recruitment Flowchart

This supplemental material has been provided by the authors to give readers additional information about their work.

© 2023 Labban M et al. *JAMA Network Open.*

eTable. Survey-Weighted Multivariable Linear Regression for Additional Travel Time (minutes) and Distance (miles) Associated With Use of Public Transportation vs Private Vehicle From Trip Originating in the Urban Setting for Health Care Visits Reported by Household Income (<$25,000; $25,000-$49,999; $50,000-$99,999; and ≥ $100,000) Accounting for Respondent, Trip, and Community Characteristics

**TRAVEL TIME (MINUTES)**								
**Additional travel burden associated with public transportation**	**<$25,000** N= 2,830 trips (WE= 1,192,038,241)		**$25,000-$49,999** N= 3,360 trips (WE= 1,121,140,466)		**$50,000-$99,999** N= 4,734 trips (WE= 1,152,528,980)		**≥ $100,000** N= 3,887 trips (WE= 1,169,812,670)	
	**B 95%CI**	**P_{int}**	**B 95%CI**	**P_{int}**	**B 95%CI**	**P_{int}**	**B 95%CI**	**P_{int}**
NHW	27.2 (8.8; 45.6)	0.90	**29.6 (20.4; 38.8)**	**<0.01**	**26.7 (17.4; 36.0)**	**<0.01**	**33.7 (12.6; 54.8)**	**< 0.01**
NHB	37.0 (-0.30; 75.5)		**85.2 (49.8; 120.6)**		-21.2 (-48.4; 6.1)		3.5 (-59.6; 66.7)	
Hispanic	29.6 (12.5; 46.8)		**26.9 (15.5; 38.3)**		**67.4 (30.5; 104.3)**		**-8.4 (-15.5; -1.2)**	
Other	25.4 (-3.2; 54.0)		9.0 (-1.5;19.5)		**63.7 (56.2; 71.1)**		**54.9 (34.0; 75.9)**	
**TRAVEL DISTANCE (MILES)**								
NHW	-2.7 (-5.8; 0.4)	0.53	0.6 (-3.7; 4.9)	0.08	-1.4 (-3.5; 0.8)	0.25	5.5 (-3.6; 14.6)	**< 0.01**
NHB	3.0 (-6.4; 12.4)		4.5 (-3.8; 12.8)		-4.3 (-12.3; 3.8)		5.7 (-15.0; 26.4)	
Hispanic	-1.8 (-5.2; 1.6)		-4.9 (-8.6; -1.1)		3.1 (-3.4; 9.6)		**-7.1 (-11.4; -2.9)**	
Other	-2.8 (-8.0; 2.5)		-3.3 (-7.7; 1.1)		2.9 (-1.6; 7.4)		2.4 (-3.5; 8.3)	

Model was adjusted for gender, race/ethnicity, age group, educational attainment, household income, whether the trip was made on business days and business hours, region in the United States, whether the place of residence was close to railway, and the proportion of renters in the neighborhood.

© 2023 Labban M et al. *JAMA Network Open.*

eFigure. Respondent Recruitment Flowchart

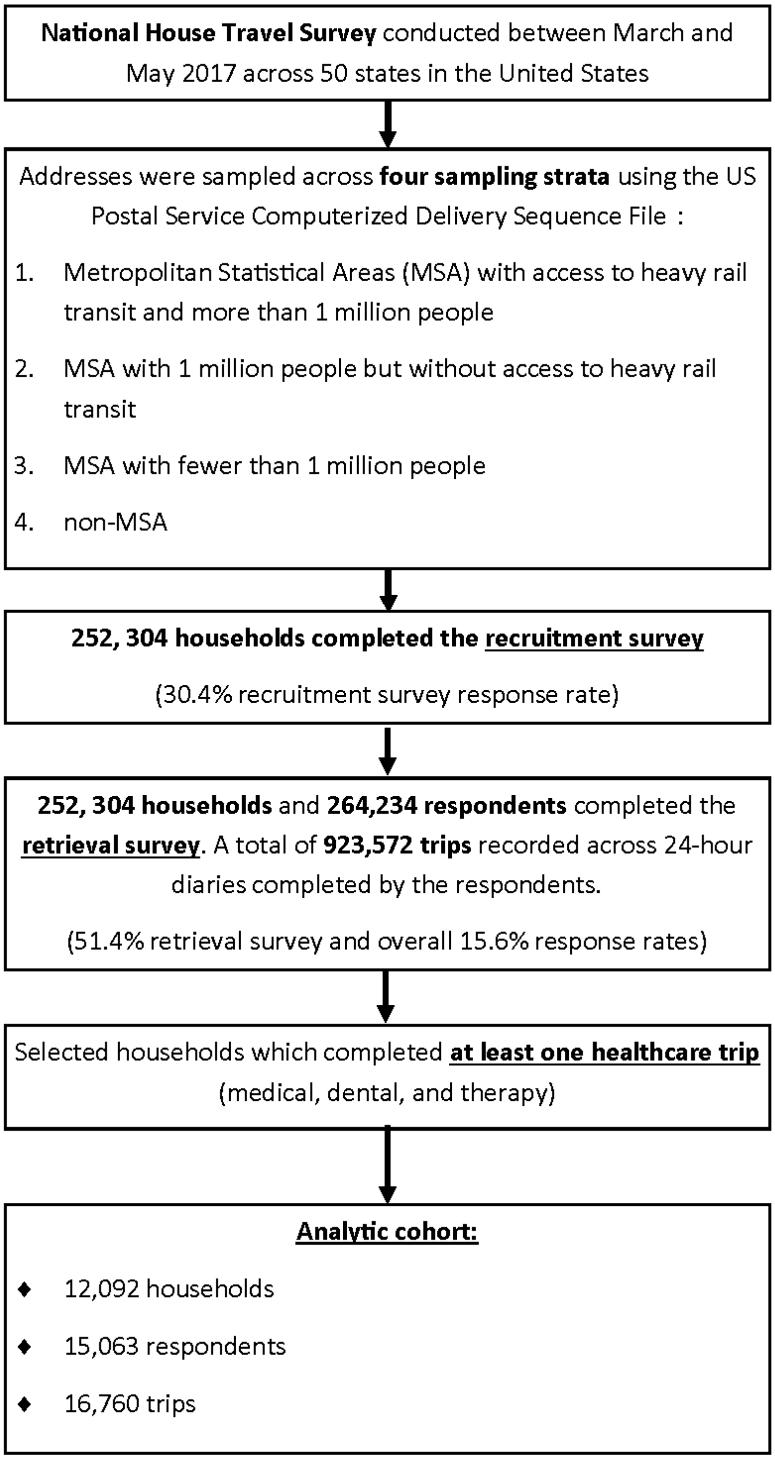

© 2023 Labban M et al. *JAMA Network Open.*
