# Supplementary material for: Disparities in Travel-Related Barriers to Accessing Health Care From the 2017 National Household Travel Survey
Source: JAMA Netw Open. 2023 Jul 27;6(7):e2325291. doi: 10.1001/jamanetworkopen.2023.25291 (PMC10375305; doi:10.1001/jamanetworkopen.2023.25291)
Supplement: Supplement 2. — Data Sharing Statement [file jamanetwopen-e2325291-s002.pdf]

Data Sharing Statement

Labban. Disparities in Travel-Related Barriers to Accessing Health Care From the 2017 National Household Travel Survey. *JAMA Netw Open*. Published July 27, 2023. doi:10.1001/jamanetworkopen.2023.25291

Data
Data available: No

Additional Information
Explanation for why data not available: The data is publicly available on the U.S. Department of Transportation Federal Highway Administration https://nhts.ornl.gov/. The codes and output are available from the corresponding author on reasonable request.
